# The complete mitochondrial genome of *Thryssa hamiltonii* and phylogenetic analysis of Engraulidae (Clupeiformes; Clupeoidei)

**DOI:** 10.1080/23802359.2018.1467232

**Published:** 2018-04-27

**Authors:** Pengfei Wang, Chao Zhao, Sigang Fan, LuLu Yan, Lihua Qiu

**Affiliations:** a Key Laboratory of South China Sea Fishery Resources Exploitation & Utilization, Ministry of Agriculture, South China Sea Fisheries Research Institute, Chinese Academy of Fishery Sciences, Guangzhou, PR China;; b Guangdong Provincial Key Laboratory of Fishery Ecology and Environment, Guangzhou, PR China

**Keywords:** Mitochondrial genome, *Thryssa hamiltonii*, Engraulidae, phylogenetic analysis

## Abstract

This study aimed to elucidate the complete mitochondrial genome (mitogenome) of *Thryssa hamiltonii* (Clupeiformes: Engraulidae). The circular mitogenome is 16,737-bp-long, including 13 protein-coding genes, 22 tRNA genes, two rRNA genes, and a non-coding control region as observed in other vertebrates. The overall base composition is as follows: A, 30.69%; T, 24.86%; C, 28.17%; G, 16.29%; a slight A + T bias of 55.55%. Phylogenetic analysis of 16 species in family Engraulidae revealed that *T. hamiltonii* clustered in subfamily Engraulinae and is closely related to *Lycothrissa crocodilus*. The present data will contribute to future phylogenetic studies on members of family Engraulidae and conservation strategies for *T. hamiltonii*.

*Thryssa hamiltonii*, family Engraulidae, is distributed throughout the tropical western Indo-Pacific region and plays an important role in marine biodiversity and recovery of fishery resources; its deteriorating resources have attracted increasing attention (Qin et al. 2011). The mitochondrial genome is an effective tool for species identification, molecular taxonomy, and population genetic analyses (Galtier et al. 2009). Therefore, the complete mitochondrial genome of *T. hamiltonii* and its phylogenetic relationships within Engraulidae were investigated in this study. The results of this study may contribute to phylogenetic analyses of the members of family Engraulidae and further conservation strategies for *T. hamiltonii*.

The specimen was collected from the South China Sea (21°88′N, 113°18′E). Muscle was sampled and frozen in liquid nitrogen and then stored at −80 °C in South China Sea Fisheries Research Institute, Guangzhou, China. Mitochondrial DNA (mtDNA) was isolated using a Mitochondrial DNA Isolation Kit (Haling Biotech Shanghai, Co., Ltd, Shanghai, China). Whole-genome sequencing was performed using the Illumina HiSeq 2500 Sequencing System (Illumina, Inc). Clean data were assembled by the SOAPdenovo Assembler program (v.2.04) and GapCloser (v.1.12) (Beijing Genomics Institute, China), and the assembled mtDNA was assessed via polymerase chain reaction. The tRNA genes were identified using tRNAscan-SE 2.0 (Lowe & Chan, 2016). Maximum Likelihood (ML) trees were constructed using MEGA6.06 software based on complete mitochondrial genomes of species in family Engraulidae (Tamura et al. 2013).

The complete mitochondrial genome of *T. hamiltonii* (GeneBank accession number KX096870) is 16,737-bp-long and contains 13 protein-coding genes (PCGs), 22 tRNA genes, 2 rRNA genes, and a control region (D-loop) (Table 1). The overall base composition of the mitogenome is the following: A, 30.69%; T, 24.86%; C, 28.17%; G, 16.29% G; a slight A + T bias of 55.55%, conserved in other species in family Engraulidae (Wang et al. 2015; Zhang and Gao 2016). Most genes are encoded on the heavy strand, except *ND6* and eight tRNA genes (*tRNA-Gln*, *tRNA-Ala*, *tRNA-Asn*, *tRNA-Cys*, *tRNA-Tyr*, *tRNA-Ser*^(^*^UCA^*^)^, *tRNA-Glu*, and *tRNA-Pro*), which are encoded on the light strand. All PCGs initiate with ATG as the start codon, except for *COX1* (GTG). This is common in vertebrate mtDNA (Wang et al. 2018). Five PCGs (*ND1*, *COX1*, *ATPase8*, *ATPase6*, and *ND4L*) typically terminate with TAA as the stop codon; however, *ND6* ends with AGG. Five PCGs (*ND2*, *COX2*, *ND3*, *ND4*, and *Cyt b*) end with T–, and *ND5* ends with TA–. Twenty-two tRNA genes, 66–76 bp, display a typical clover-leaf secondary structure, except for *tRNA-Ser*^(^*^AGC^*^)^. The control region, with high A + T content (61.19%), is located between *tRNA-Pro* and *tRNA-Phe*.

**Table 1.** Features of the mitochondrial genome of *Thryssa hamiltonii*.

	Position		Size (bp)	Amino	Codon			
Gene	From	To	Nucleotide	acid	Start	Stop	Gap	Strand
*tRNA-Phe*	1	69	69				0	H
*12S rRNA*	70	1022	953				0	H
*tRNA-Val*	1023	1094	72				0	H
*16S rRNA*	1095	2783	1688				0	H
*tRNA-Leu*^(^*^UUA^*^)^	2784	2859	76				0	H
*ND1*	2860	3834	975	324	ATG	TAA	0	H
*tRNA-Ile*	3836	3907	72				1	H
*tRNA-Gln*	3907	3977	71				−1	L
*tRNA-Met*	3977	4045	69				−1	H
*ND2*	4046	5090	1045	348	ATG	T–	0	H
*tRNA-Trp*	5091	5162	72				0	H
*tRNA-Ala*	5165	5233	69				2	L
*tRNA-Asn*	5235	5307	73				1	L
*tRNA-Cys*	5339	5404	66				31	L
*tRNA-Tyr*	5405	5475	71				0	L
*COX1*	5477	7021	1545	514	GTG	TAA	1	H
*tRNA-Ser*^(^*^UCA^*^)^	7022	7092	71				0	L
*tRNA-Asp*	7098	7166	69				5	H
*COX2*	7178	7868	691	230	ATG	T–	11	H
*tRNA-Lys*	7869	7942	74				0	H
*ATP8*	7944	8111	168	55	ATG	TAA	1	H
*ATP6*	8102	8785	684	227	ATG	TAA	−10	H
*COX3*	8785	9569	785	261	ATG	TA-	−1	H
*tRNA-Gly*	9570	9641	72				0	H
*ND3*	9642	9900	349	116	ATG	T–	0	H
*tRNA-Arg*	9991	10,059	69				0	H
*ND4L*	10,060	10,356	297	98	ATG	TAA	0	H
*ND4*	10,350	11,730	1381	460	ATG	T–	−6	H
*tRNA-His*	11,731	11,799	69				0	H
*tRNA-Ser*^(^*^AGC^*^)^	11,801	11,867	67				1	H
*tRNA-Leu*^(^*^CUA^*^)^	11,868	11,939	72				0	H
*ND5*	11,940	13,774	1835	611	ATG	TA-	0	H
*ND6*	13,771	14,292	522	173	ATG	AGG	−4	L
*tRNA-Glu*	14,293	14,361	69				0	L
*Cyt b*	14,366	15,506	1141	380	ATG	T–	4	H
*tRNA-Thr*	15,507	15,576	70				0	H
*tRNA-Pro*	15,576	15,647	72				−1	L
*D-loop*	15,648	16,737	1090				0	H

To elucidate the phylogenetic position of *T. hamiltonii*, a ML phylogenetic tree was constructed with 16 species in family Engraulidae based on their complete mitogenome (Figure 1); it clearly shows that the species in family Engraulidae are divided into two subfamilies, Engraulinae and Coilinae, and *T. hamiltonii* is clustered in subfamily Engraulinae and closely related to *Lycothrissa crocodilus*. The present data may potentially contribute to taxonomic and phylogenetic studies of members of the family Engraulidae and further conservation strategies for *T. hamiltonii*.

**Figure 1.**
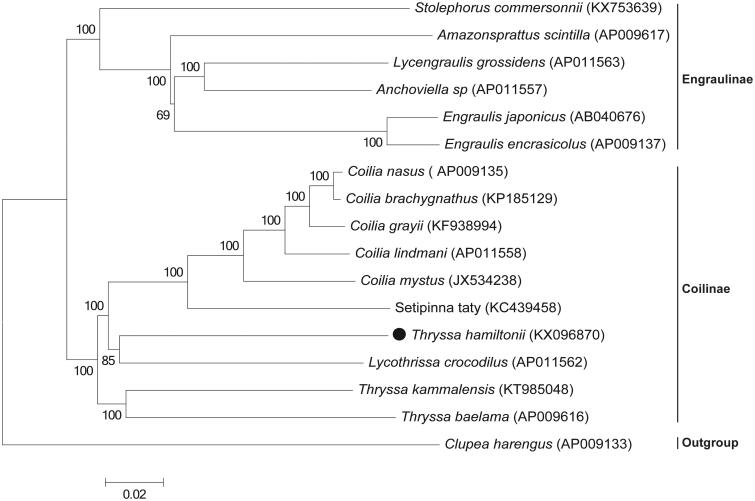
Phylogenetic analysis of 16 species of family Engraulidae based on their complete mitochondrial genomes, determined using the Neighbour-joining method. Bootstrap support values (1000 replicates) are indicated at the nodes. The number after the species name is the GenBank accession number.
